# Dairy consumption, motivations, and nutritional status among schoolchildren in northwestern Morocco: socioeconomic factors and place of residence

**DOI:** 10.3389/fnut.2026.1831350

**Published:** 2026-06-17

**Authors:** Hasna Attaf, Dalal Ayyad, Fatima-Zahra Yassif, Firdaous Chairat, Abdessamad Jalouni, Samir Bikri, Youssef Aboussaleh

**Affiliations:** 1 Laboratory of Biology and Health, Faculty of Sciences, Ibn Tofail University, Kenitra, Morocco; 2 Laboratory of Science and Engineering Research, Faculty of Sciences and Technologies, Sidi Mohamed Ben Abdellah University, Fez, Morocco

**Keywords:** consumption behavior, dairy consumption, linear growth, Morocco, nutritional status, schoolchildren, socioeconomic factors, urban-peri-urban differences

## Abstract

Morocco, like many low and middle-income countries, is undergoing a rapid nutritional transition marked by the concurrent persistence of stunting and the emergence of overweight and obesity, a phenomenon known as the double burden of malnutrition (DBM). Dairy products are recognized as nutrient-dense foods that support linear growth through their role in insulin-like growth factor-1 (IGF-1) stimulation; however, dairy intake patterns and their relationship with nutritional status among Moroccan schoolchildren remain poorly documented, particularly across urban-peri-urban gradients. This cross-sectional study was conducted as part of the SUPREM-MILK project, Work Package 1 (WP1). A total of 248 schoolchildren aged 7–14 years enrolled in pilot public primary schools located in urban and peri-urban public primary schools in Kenitra province, northwestern Morocco (January–June 2025). Dairy product consumption frequency was assessed using a Food Frequency Questionnaire (FFQ). Nutritional status was evaluated through anthropometric measurements, including height-for-age Z-scores (HAZ), BMI-for-age Z-scores (BAZ), and waist-to-height ratio (WHtR). Liquid milk was the most frequently consumed dairy product among the children surveyed (33.9% reported high consumption; 56.5% moderate), while traditional dairy products such as Lben and Jben were consumed less frequently. Taste preference was the dominant motivation for dairy intake across both residential settings. Urban children showed significantly higher HAZ (0.19 ± 1.23 vs. −0.43 ± 0.96; *p* < 0.001) and BAZ (0.51 ± 1.14 vs. 0.01 ± 0.97; *p* < 0.001) than peri-urban peers, yet abdominal obesity was markedly more prevalent in urban areas (16.8% vs. 2.4%; *p* < 0.001). Household income (*p* = 0.006) and parental education (*p* < 0.01) were significant determinants of linear growth. After full covariate adjustment, no independent association was detected between dairy consumption frequency and the risk of overweight, stunting, or abdominal obesity (all *p* > 0.05). These findings indicate a context-specific nutritional transition in which urbanization is associated with improved linear growth but a higher risk of abdominal obesity. The absence of an independent association between dairy consumption frequency and nutritional outcomes after full covariate adjustment reflects the complexity of the diet growth relationship in this context. Differentiated public health strategies are therefore needed: promoting balanced diets in urban settings and improving dietary diversity in peri-urban areas, to address this dual nutritional burden.

## 1 Introduction

Malnutrition, in all its forms, remains one of the most critical public health challenges affecting the pediatric population worldwide (1, 2). It is estimated to contribute directly or indirectly to nearly half of all child mortality, with undernutrition alone accounting for more than one-third of these fatalities (3, 4). In low and middle-income countries (LMICs), stunting remains a major concern, affecting millions of children due to inadequate nutritional intake and suboptimal sanitary conditions (5, 6). In Africa, this burden remains particularly severe, with stunting rates reaching alarming levels in several regions, including sub-Saharan and Mediterranean areas (7, 8).

Concurrently, LMICs are experiencing an accelerating nutritional transition driven by urbanization and globalization (9, 10). This transition is characterized by a shift away from traditional dietary patterns toward so-called “Westernized” diets, marked by the widespread availability of ultra-processed foods that are energy-dense but poor in essential nutrients (11, 12). This profound transformation is contributing to the emergence of the double burden of malnutrition (DBM), a paradoxical situation in which undernutrition (stunting and thinness) and overnutrition (overweight and obesity) coexist within the same population, or even within the same household (7, 13). In Morocco, recent studies have shown that overweight and obesity are now more prevalent than undernutrition among certain groups of urban schoolchildren (14).

Urbanization is progressively reshaping food environments, generating significant disparities between dense urban centers and peri-urban areas (15, 16). While rural regions have historically been the primary focus of nutritional interventions, peri-urban areas are increasingly recognized as emerging zones of vulnerability. These areas are often characterized by precarious infrastructure, growing dependence on purchased food, and persistent food insecurity (17). In this context, anthropometry remains one of the most practical and indispensable tools for assessing children’s well-being and identifying inequalities in human development (18).

Within this complex nutritional landscape, milk and dairy products occupy a strategically important position. Recognized as nutrient-dense foods, they represent an important source of high-quality proteins, highly bioavailable calcium, and essential micronutrients such as vitamins A, D, and B12 (19, 20). The so-called “milk hypothesis” proposes that dairy consumption plays a key role in human physical development (21). This relationship is biologically mediated by the stimulation of insulin-like growth factor-1 (IGF-1), which directly acts on the epiphyseal growth plates to regulate bone growth (22, 23). Large-scale studies, such as the SEANUTS surveys conducted in Southeast Asia, have provided strong evidence of a positive association between regular dairy consumption and improved height-for-age Z-scores (HAZ), thereby significantly reducing the risk of stunting (24, 25).

Despite these documented benefits, dairy consumption in Morocco remains well below international recommendations (14, 26). Intake levels are strongly influenced by socioeconomic factors, particularly household income and parental education (16, 27). Paternal education, in particular, appears to be an important structural determinant influencing access to high-quality animal protein (1, 28). At the same time, the Moroccan dairy sector is undergoing structural transformation: industrial processing increasingly dominates the liquid milk market, whereas traditional fermented products such as Lben and Raib are gradually becoming limited to marginal or culturally specific consumption, although they continue to maintain a strong identity in peri-urban communities (16, 17). This divergence in product preferences across residential settings may have important nutritional implications that have yet to be fully characterized.

Another dimension that remains insufficiently considered in nutrition policies is the behavioral and cognitive aspect of food choices. Among school-aged children, dietary preferences are primarily driven by sensory factors such as taste rather than by awareness of health benefits (14, 29). Understanding these behavioral determinants is essential for designing effective nutrition education interventions. Beyond physical growth, adequate nutrient intake, including the B-vitamins supplied by dairy products, has been associated with improved cognitive function, attention, and academic performance (30–32). At the same time, food insecurity and iron deficiency have been identified as major barriers to adolescents’ educational trajectories (33).

Despite these challenges, data specifically linking dairy consumption patterns (industrial versus traditional products) to children’s nutritional status, while accounting for the urban-peri-urban residential gradient, remain scarce in Morocco. Nutritional deficiencies are widespread: 85.5% of schoolchildren in Rabat have been reported to have insufficient calcium intake (26); vitamin A deficiency affects more than half of children in certain oasis regions (28); and iron-deficiency anemia remains a major public health concern across Moroccan schools (14, 34). A critical unresolved question is whether urbanization, while potentially protecting against stunting through improved food access, may simultaneously accelerate the emergence of abdominal obesity in this nutritional transition context.

Kenitra province, in northwestern Morocco, was selected as the study setting because its rapid urbanization creates a within-region urban-peri-urban gradient uniquely suited for examining concurrent undernutrition and emerging overweight within a single administrative context (15, 16). Our team’s prior work in this province has documented high rates of iron-deficiency anemia and suboptimal dietary diversity among local schoolchildren (14, 18), establishing both the epidemiological need and the institutional foundation, through Ibn Tofaïl University and the SUPREM-MILK project, for the present investigation.

This cross-sectional study aimed to characterize dairy consumption patterns among schoolchildren aged 7–14 years in Kenitra province, northwestern Morocco, and to examine how these patterns vary by residential setting and socioeconomic status. Three specific objectives were pursued: (1) to describe the frequency and types of dairy products consumed; (2) to identify socioeconomic and demographic determinants of dairy intake; and (3) to assess the association between dairy consumption frequency and nutritional status indicators, including HAZ, BAZ, and abdominal obesity (WHtR). The findings are intended to inform the design of context-sensitive public health and nutrition education interventions addressing both the persistent burden of undernutrition and the emerging challenge of childhood obesity in Morocco.

## 2 Materials and methods

### 2.1 Data collection and study area

**Figure 1:**
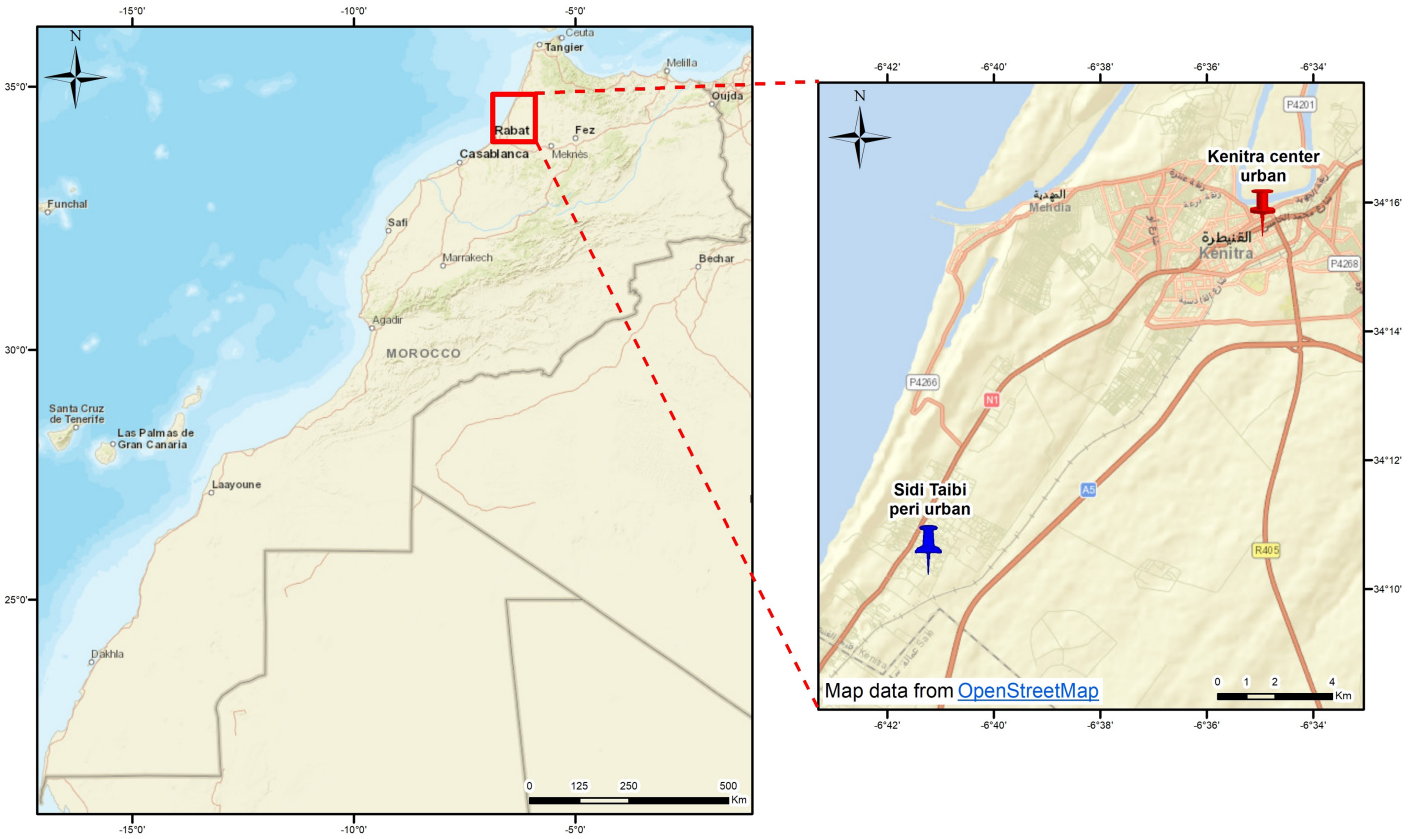
This map is licensed under Open Data Commons Open Database License (ODbL) by the Open Street Map Foundation (OSMF).

This study was conducted between January and June 2025 among schoolchildren attending public primary schools in Kenitra Province, northwestern Morocco (Figure 1). The study area encompasses both urban and peri-urban municipalities characterized by a marked gradient in socioeconomic conditions and food environments. Data collection was carried out within the framework of the SUPREM-MILK project, an international research initiative funded by the PRIMA Foundation under its Section 2 caLL, specifically its Work Package 1 (WP1), which focuses on understanding dietary behaviors and consumer practices related to dairy products among schoolchildren.

A total of 248 schoolchildren aged 7–14 years were enrolled. Schools were purposively selected to reflect the urban-peri-urban gradient; within each school, one class per grade level (Grades 2 to 5) was randomly selected to ensure balanced age-group representation. Participation was voluntary, and written informed consent was obtained from all parents or legal guardians before data collection. Data were collected through structured face-to-face interviews using a standardized questionnaire developed from tools commonly used in nutritional epidemiology and adapted to the Moroccan sociocultural context. Before the main survey, the instrument was pilot-tested among 52 children with comparable sociodemographic characteristics; minor adaptations were made on the basis of pilot feedback, and pilot data were excluded from the final analysis.

### 2.2 Study design and dietary assessment

The questionnaire comprised six thematic sections: socioeconomic and household characteristics; anthropometric data; dairy product consumption frequency and motivations; general dietary habits and nutritional intake; lifestyle-related factors (physical activity, screen time, and sleep duration); and general health status and academic performance. The last three sections were collected in the context of the broader SUPREM-MILK project objectives and will be reported in separate publications. The present study draws exclusively on sections 1 (socioeconomic characteristics), 2 (anthropometric data), and 3 (dairy consumption frequency and motivations).

Sociodemographic variables collected included age, sex, residential area (urban or peri-urban), household size, household income level, and parental education level for both mother and father. Dairy consumption was assessed using a semi-quantitative Food Frequency Questionnaire (FFQ) covering seven products: milk, yogurt, cheese, butter, and the traditional fermented products *Lben*, *Jben*, and *Raib*, spanning two distinct segments of the Moroccan dairy market, industrial (milk, yogurt, cheese) and traditional or artisanal (butter, *Lben*, *Jben*, *Raib*), a distinction with both nutritional and cultural relevance in the context of Morocco’s ongoing dietary transition. Children were asked to report their usual frequency of consumption of each product over the preceding week. A seven-day reference period was selected to balance ecological validity with the age group’s recall capacity, thereby minimizing memory bias while capturing habitual intake patterns. Consumption frequency was operationalized as an ordinal variable. It was classified *a priori* into three categories consistent with comparable FFQ-based pediatric studies (2, 3): Low (≤1 time per week, including no consumption), Moderate (2–4 times per week), and High (≥5 times per week, including daily). Participants were additionally asked to identify their primary motivation for consuming dairy products, distinguishing between taste preference and perceived health benefits.

Inclusion criteria were: (1) enrolment in one of the selected public primary schools; (2) age between 7 and 14 years; (3) apparently good general health status as assessed by the class teacher; and (4) provision of written parental or legal-guardian consent. Children enrolled in Grade 1 and Grade 6 were excluded to ensure linguistic comprehension homogeneity across participants. Children who were absent on the day of data collection or whose parents declined to provide consent were also excluded.

### 2.3 Anthropometric measurements

Anthropometric measurements were conducted according to standardized procedures (35). Body weight was measured using a TANITA electronic scale with a precision of 0.1 kg, with children wearing light clothing and no shoes. Height was measured using a portable stadiometer, accurate to 0.1 cm, with children standing upright, feet together, and head positioned according to the Frankfurt plane. Weight and height measurements were taken twice, and the mean value was used for statistical analysis. Waist circumference was measured using a non-stretchable measuring tape, positioned horizontally midway between the lowest rib and the iliac crest. Mid-upper arm circumference (MUAC) was measured on the left arm, midway between the acromion and the olecranon processes. MUAC and waist circumference were each recorded once per child and used as complementary indicators of nutritional status. All measurements were performed by the same trained investigators throughout the data collection period to ensure consistency and minimize inter-observer variability.

### 2.4 Nutritional status indices

Body mass index (BMI) was calculated using the formula.

$$\mathrm{BMI}=\text{weight}\;\underline{\left(\mathrm{kg}\right)}\;\text{heigh}t^{2}(m^{2})$$

BMI-for-age Z-score (BAZ) and height-for-age Z-score (HAZ) were calculated using WHO AnthroPlus software, based on the World Health Organization growth reference for children and adolescents aged 5–19 years (36).

Nutritional status was classified according to the following WHO-standardized thresholds:

- Stunting: HAZ < −2 SD.
- Overweight/obesity: BAZ > +1 SD.
- Abdominal obesity: Waist-to-height ratio (WHtR) ≥ 0.5.

These anthropometric indices were used as the primary outcomes of interest and were analyzed in relation to dairy consumption frequency, residential area, and sociodemographic characteristics.

### 2.5 Statistical analysis

Data were cleaned and analyzed using IBM SPSS Statistics version 25 and Python version 3.14. Before analysis, the normality of continuous variables was assessed using the Shapiro–Wilk test. Categorical variables are presented as frequencies and percentages (*n*, %), whereas continuous variables are expressed as mean ± standard deviation (SD) or median with interquartile range (IQR), depending on the distribution of the data. Given that most continuous variables did not follow a normal distribution, group comparisons were performed using non-parametric tests: the Mann–Whitney *U* test for comparisons between two independent groups (urban vs. peri-urban), and the Kruskal–Wallis test for comparisons across more than two groups (e.g., income or education categories). Differences in proportions for categorical variables were assessed using the Pearson chi-square test, as appropriate. Associations between dairy consumption frequency and nutritional outcomes were examined in two complementary steps. First, multiple linear regression models were used to assess associations between dairy product’s consumption frequency (ordinal predictor: Low, Moderate, High, with Low as reference) and continuous anthropometric outcomes (BMI, BAZ, and HAZ). Second, binary logistic regression models examined associations between dairy product’s consumption frequency and binary nutritional outcomes (overweight/obesity, stunting, and abdominal obesity). All models were adjusted for the following potential confounders selected *a priori* based on the literature: age group, sex, residential area, household size, household income level, and parental education level for both mother and father. Results from logistic models are reported as adjusted odds ratios (aOR) with 95% confidence intervals (95% CI). For outcomes with sparse events (cell counts < 5), Firth’s penalized-likelihood logistic regression was applied to obtain stable estimates. The Hosmer–Lemeshow test was used to assess the goodness of fit for each logistic model. A two-sided *p*-value < 0.05 was considered statistically significant.

### 2.6 Ethical approval

The studies involving human participants were reviewed and approved by the institutional ethics committee of the Department of Biology, Laboratory of Biology and Health, Ibn Tofail University, Kenitra, Morocco. The studies were conducted in accordance with the local legislation and institutional requirements. Written informed consent was obtained from the parents or legal guardians of all participating children before their inclusion in the study.

## 3 Results

### 3.1 Sociodemographic characteristics and anthropometric profile of the study population

A total of 248 schoolchildren were included in this study, comprising 125 urban (50.4%) and 123 peri-urban (49.6%) participants, with a near-equal sex distribution (53.2% boys, 46.8% girls). The majority of children (72.2%) were aged 7–10 years, and the remaining 27.8% fell within the 11–14 year age group. No significant differences were observed between residential areas for age group (*p* = 0.937) or sex (*p* = 0.993), indicating adequate comparability of the two groups on these baseline characteristics (Table 1).

**Table 1:** Descriptive characteristics of the study population overall and by residential area.

Variable	Total (*n* = 248)	Urban (*n* = 125)	Peri-urban (*n* = 123)	*p*
Socioeconomic and household characteristics				
Age group, *n* (%)				
7–10 years	179 (72.2)	91 (72.8)	88 (71.5)	0.937
11–14 years	69 (27.8)	34 (27.2)	35 (28.5)	
Sex, *n* (%)				
Boys	132 (53.2)	66 (52.8)	66 (53.7)	0.993
Girls	116 (46.8)	59 (47.2)	57 (46.3)	
Household size, *n* (%)				
≤5 members	150 (60.5)	95 (76.0)	55 (44.7)	<0.001***
>5 members	98 (39.5)	30 (24.0)	68 (55.3)	
Household income^†^, *n* (%)				
Low	202 (81.5)	88 (70.4)	114 (92.7)	<0.001***
Mid/high	46 (18.5)	37 (29.6)	9 (7.3)	
Mother’s education, *n* (%)				
Illiterate	23 (9.3)	12 (9.6)	11 (8.9)	<0.001***
Primary	131 (52.8)	32 (25.6)	99 (80.5)	
Secondary	88 (35.5)	75 (60.0)	13 (10.6)	
University	6 (2.4)	6 (4.8)	0 (0.0)	
Father’s education, *n* (%)				
Illiterate	10 (4.0)	0 (0.0)	10 (8.1)	<0.001***
Primary	148 (59.7)	64 (51.2)	84 (68.3)	
Secondary	35 (14.1)	16 (12.8)	19 (15.4)	
University	55 (22.2)	45 (36.0)	10 (8.1)	
Anthropometric outcomes and nutritional categories				
BMI (kg/m^2^), mean ± SD	17.14 ± 2.65	17.67 ± 2.88	16.61 ± 2.28	<0.001***
BAZ (z-score), mean ± SD	0.26 ± 1.09	0.51 ± 1.14	0.01 ± 0.97	<0.001***
HAZ (z-score), mean ± SD	−0.12 ± 1.14	0.19 ± 1.23	−0.43 ± 0.96	<0.001***
Abdominal obesity (WHtR ≥ 0.5), *n* (%)	24 (9.7)	21 (16.8)	3 (2.4)	<0.001***
Overweight/obesity (BAZ > +1 SD), *n* (%)	55 (22.2)	38 (30.4)	17 (13.8)	0.003**
Stunting (HAZ < −2 SD), *n* (%)	9 (3.6)	4 (3.2)	5 (4.1)	0.980

BMI, body mass index; BAZ, BMI-for-age z-score; HAZ, height-for-age z-score; WHtR, waist-to-height ratio; SD, standard deviation.

*p < 0.05; **p < 0.01; ***p < 0.001. Bold *p*-values: statistically significant. Continuous: mean ± SD; categorical: *n* (%).

^†^Household income recategorized as low vs. middle/high due to a very small high-income group.

Household size differed significantly between areas (*p* < 0.001): urban households were predominantly small (≤5 members: 76.0%), whereas peri-urban households were more likely to be large (>5 members: 55.3%). A similarly marked contrast was found for household income (*p* < 0.001): low-income households were substantially more prevalent in peri-urban areas (92.7%) compared to urban areas (70.4%), while middle/high-income households were nearly four times more frequent in urban settings (29.6% vs. 7.3%) (Table 1).

Parental education levels differed significantly between residential areas for both mothers (*p* < 0.001) and fathers (*p* < 0.001). Urban mothers were predominantly educated to secondary level (60.0%), whereas peri-urban mothers were largely at primary level (80.5%), with no peri-urban mothers holding university-level education. Among fathers, university education was markedly more common in urban areas (36.0% vs. 8.1%), while illiteracy was absent among urban fathers (0.0%) but represented 8.1% of peri-urban fathers. These findings confirm a marked socioeconomic gradient between the two residential settings, which must be accounted for in all subsequent analyses (Table 1).

Significant differences between urban and peri-urban children were found across all continuous anthropometric indicators. Urban children exhibited a significantly higher mean BMI (17.67 ± 2.88 vs. 16.61 ± 2.28 kg/m^2^; *p* < 0.001), higher BAZ (0.51 ± 1.14 vs. 0.01 ± 0.97; *p* < 0.001), and higher HAZ (0.19 ± 1.23 vs. −0.43 ± 0.96; *p* < 0.001). With respect to binary nutritional outcomes, abdominal obesity was significantly more prevalent among urban children (16.8% vs. 2.4%; *p* < 0.001), as was overweight/obesity (30.4% vs. 13.8%; *p* = 0.003). Stunting was rare and did not differ significantly between areas (3.2% vs. 4.1%; *p* = 0.980) (Table 1).

Overall, urban children show a double burden profile-better linear growth (higher HAZ) but markedly higher adiposity (abdominal obesity 16.8% vs. 2.4%), consistent with an accelerated nutritional transition in urban Morocco.

### 3.2 Dairy consumption patterns

Consumption frequency patterns varied considerably across dairy products (Table 2). Most children fell into the moderate consumption category for liquid milk (56.5%), cheese (46.0%), and yogurt (46.0%), while low consumption predominated for traditional fermented products: lben (69.4%), Jben (72.6%), and Raib (44.0%). The most frequently consumed product was milk to 27.4% for Jben, revealing a clear gradient from widely consumed industrial products to marginalized traditional fermented products. Milk was the most frequently consumed product (mean score 2.24 ± 0.62; high consumption 33.9%), followed by Cheese (83.1% participation; mean 2.20 ± 0.71) and Yogurt (76.2%; mean 2.06 ± 0.73). These three industrial dairy products were integrated into the regular diet of the majority of schoolchildren, irrespective of residential setting (Table 2).

**Table 2:** Dairy consumption patterns among schoolchildren (*n* = 248).

Product	Low^‡^, *n* (%)	Moderate, *n* (%)	High, *n* (%)	Mean score (1–3), mean ± SD	Participation (mod/high), *n* (%)
Milk	24 (9.7)	140 (56.5)	84 (33.9)	2.24 ± 0.62	224 (90.3%)
Cheese	42 (16.9)	114 (46.0)	92 (37.1)	2.20 ± 0.71	206 (83.1%)
Yogurt	59 (23.8)	114 (46.0)	75 (30.2)	2.06 ± 0.73	189 (76.2%)
Raib	109 (44.0)	73 (29.4)	66 (26.6)	1.83 ± 0.82	139 (56.0%)
Butter	113 (45.6)	41 (16.5)	94 (37.9)	1.92 ± 0.91	135 (54.4%)
Lben	172 (69.4)	10 (4.0)	66 (26.6)	1.57 ± 0.88	76 (30.6%)
Jben	180 (72.6)	31 (12.5)	37 (14.9)	1.42 ± 0.74	68 (27.4%)

Consumption frequency: Low = ≤1 time/week or never; Moderate = 2–4 times/week; High = ≥5 times/week (including daily).

^‡^“Low consumption” includes both infrequent consumers and non-consumers (children who never consume the product).

Participation = proportion of children with moderate or high consumption (binary indicator). Products ranked by descending participation rate.

In contrast, traditional fermented dairy products showed markedly lower participation: Lben (30.6%) and Jben (27.4%) were each reported as low or never consumed by over 69 and 72% of children, respectively. Butter and Raib occupied intermediate positions (participation 54.4 and 56.0%, respectively). Notably, butter presented a bimodal distribution, a high proportion of low consumers (45.6%) coexisting with a substantial proportion of high consumers (37.9%), consistent with its dual function as both a traditional food item and a daily culinary ingredient. These patterns reflect an ongoing nutritional transition in Morocco toward industrially processed dairy, though traditional products persist, particularly in peri-urban settings (see Supplementary Table S1).

### 3.3 Motivations for milk consumption according to residential area

**Figure 2:**
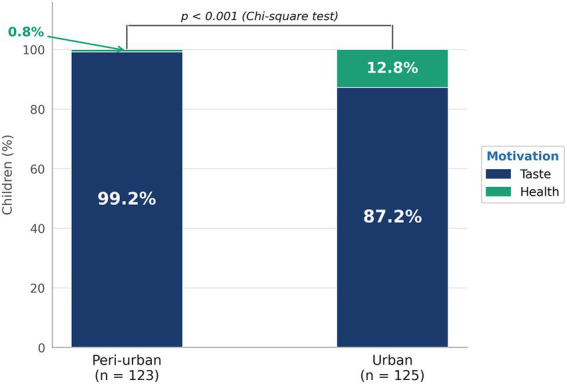
Primary motivation for milk consumption by residential area among schoolchildren in Kenitra Province, northwestern Morocco (*n* = 248). Stacked bars represent the proportion of children citing taste or health as their primary motivation within each residential group. Health-related motivation was markedly more prevalent among urban children (12.8%) than among peri-urban children (0.8%; *p* < 0.001, Chi-square test).

In addition to consumption frequency, participants were asked about their primary motivation for consuming milk. Two motivations were identified: taste and health. Taste preference was the predominant motivation in both residential areas, reported by 99.2% of peri-urban and 87.2% of urban children (Figure 2). In contrast, health-related motivation was reported by 12.8% of urban children versus only 0.8% of peri-urban children (*p* < 0.001, Pearson chi-square test), a difference suggesting that urban schoolchildren, or their families, are more likely to associate milk consumption with perceived health benefits, potentially reflecting greater exposure to nutritional education and health-promoting messages in urban environments.

### 3.4 Dairy consumption frequency and sociodemographic determinants

**Figure 3:**
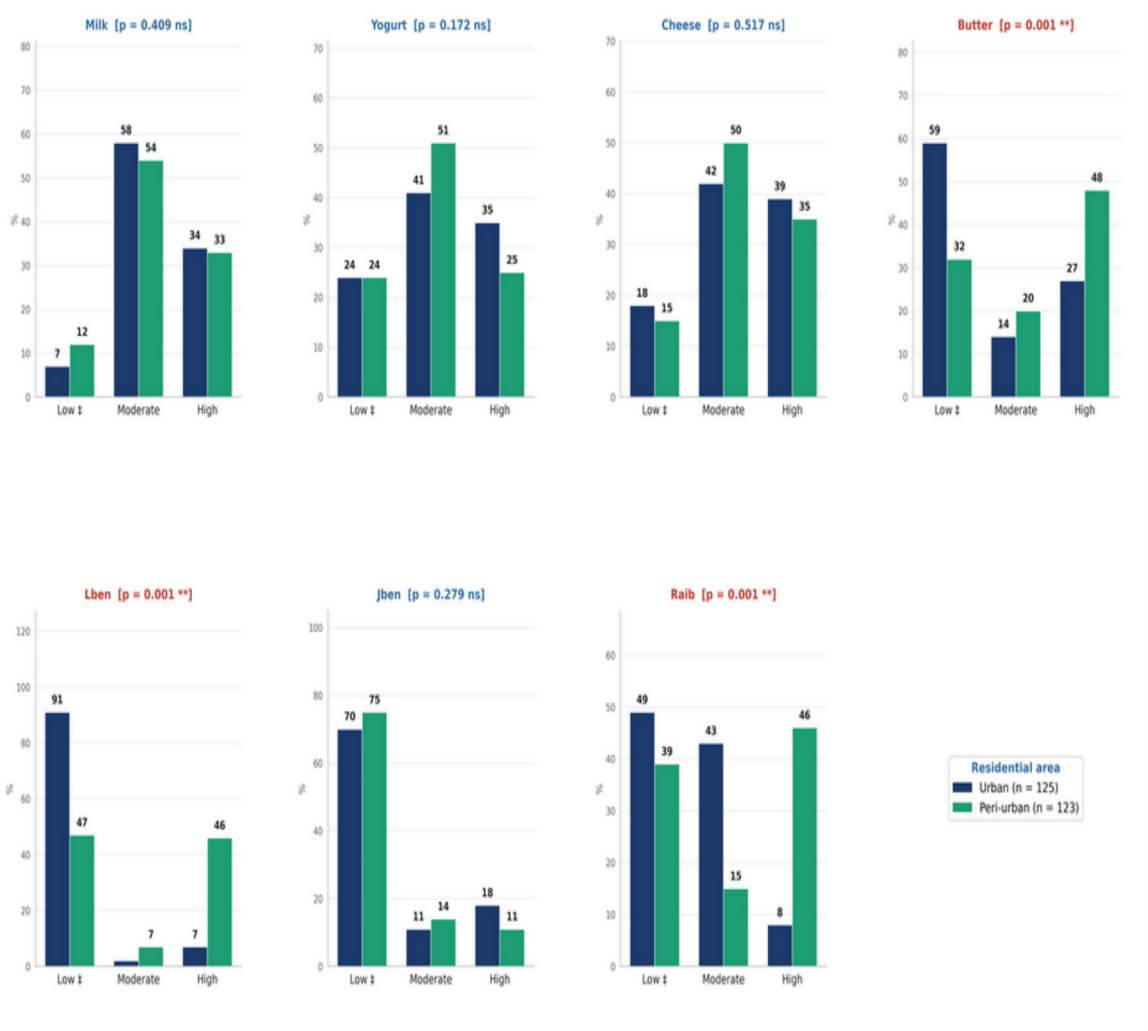
Dairy product consumption frequency by residential area (*n* = 248). Grouped bars show percentages within urban (dark blue, *n* = 125) and peri-urban (teal, *n* = 123) groups for each consumption category. ***p < 0.001; ns = not significant (Chi-square test). ^‡^Low includes non-consumers and infrequent consumers. Values labelled when ≥5%.

Residential area was the strongest sociodemographic predictor of dairy consumption frequency, with highly significant differences observed for traditional products: Butter (*p* < 0.001), Lben (*p* < 0.001), and Raib (*p* < 0.001). Peri-urban children showed substantially higher high-level consumption of Lben (46.3% vs. 7.2% in urban areas), Butter (48.8% vs. 27.2%), and Raib (45.5% vs. 8.0%). In contrast, modern dairy products, milk, cheese, and yogurt, showed comparable consumption distributions across both residential areas (all *p* > 0.05), indicating their uniform integration into children’s diets regardless of setting (Supplementary Table S1; Figure 3).

**Figure 4:**
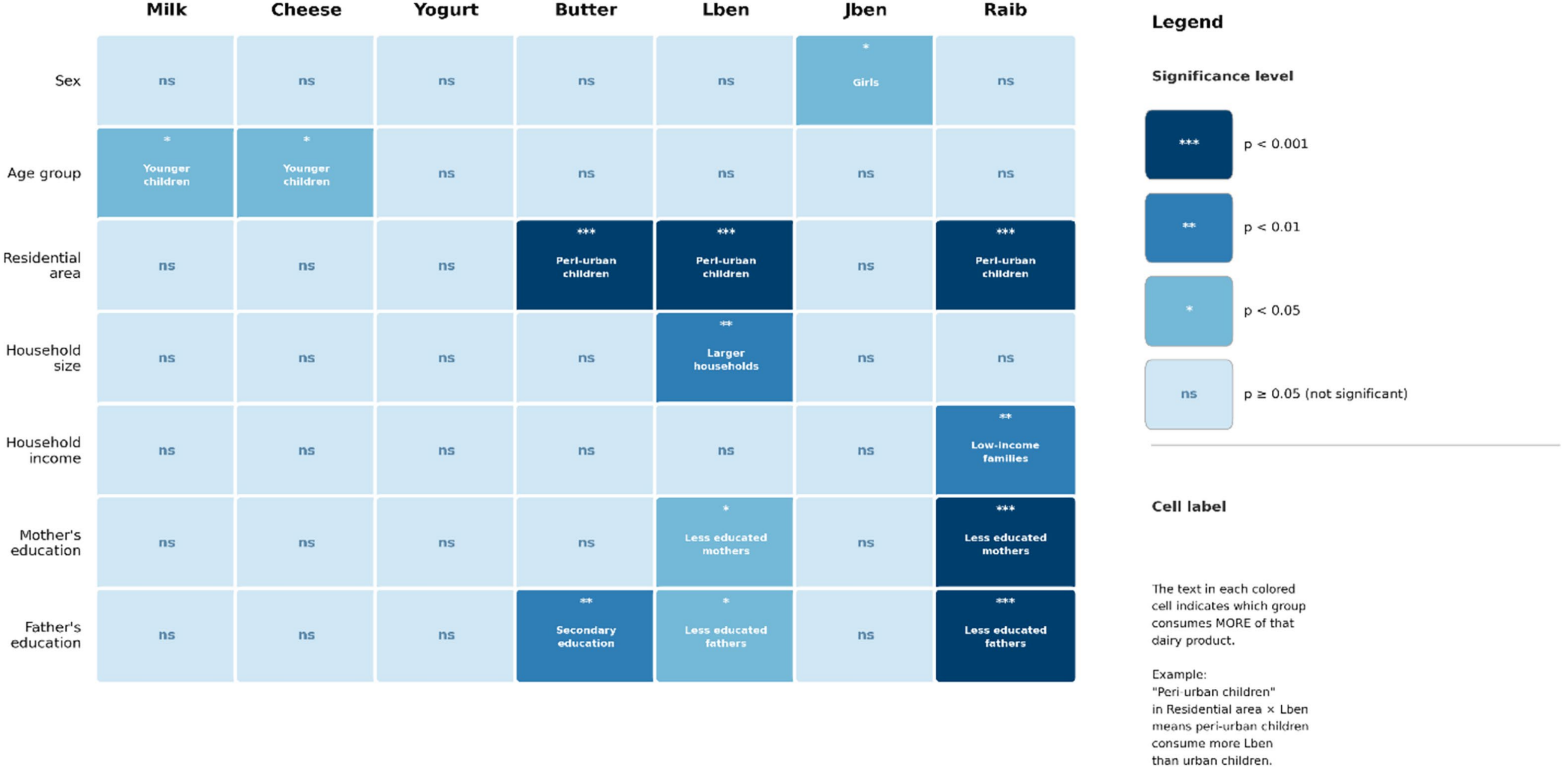
Heatmap of Chi-square test *p*-values for associations between sociodemographic factors and dairy product consumption frequency categories among schoolchildren in Kenitra province, Morocco (*n* = 248). Color intensity reflects the strength of statistical significance. The text in each significant cell indicates the group with higher consumption of the corresponding dairy product. HH = household. ^‡^Low consumption includes non-consumers. Full cross-tabulations are provided in Supplementary Tables S2a,b.

Among other sociodemographic factors, age group was significantly associated with milk (*p* = 0.021) and cheese (*p* = 0.013) consumption, with younger children (7–10 years) showing higher participation rates, consistent with a decline in dairy intake during adolescence. Household size was significantly associated with Lben consumption (*p* = 0.002), and household income with Raib (*p* = 0.002). Parental education showed inverse gradients for traditional dairy products: higher education was associated with lower consumption of Lben, Raib, and butter, while no significant education effects were detected for modern dairy products. As illustrated in Figure 4, the residential area was the strongest overall predictor of traditional dairy consumption, with highly significant associations observed for Butter, Lben, and Raib (all *p* < 0.001), followed by parental education and household size. Full cross-tabulations are provided in Supplementary Tables S2a,b.

### 3.5 Association between dairy product consumption frequency and nutritional outcomes

To provide a comprehensive evaluation of the relationship between dairy product consumption and nutritional outcomes, regression analyses were conducted for all seven dairy products assessed in this study (milk, cheese, yogurt, butter, Lben, Jben, and Raib). Adjusted linear regression models (Panel A) examined associations between consumption frequency and continuous outcomes (BMI, BAZ, and HAZ), while binary logistic regression models (Panel B) examined associations with binary outcomes (overweight/obesity, stunting, and abdominal obesity). For each product, Low consumption served as the reference category, and all models were adjusted for the same set of covariates: age group, sex, residential area, household size, household income, and parental education (mother and father). Results are presented in Tables 3, 4.

**Table 3:** Associations between dairy product consumption frequency and continuous nutritional outcomes, adjusted linear regression models (*n* = 248).

Dairy product	Outcome	Moderate vs. low (ref.)		High vs. low (ref.)	
		*β* (95% CI)	*p*	*β* (95% CI)	*p*
Milk	BMI (kg/m^2^)	−0.098 (−1.272 to 1.076)	0.870	−0.084 (−1.314 to 1.145)	0.893
	BAZ (z-score)	−0.154 (−0.638 to 0.330)	0.531	−0.121 (−0.628 to 0.386)	0.639
	HAZ (z-score)	−0.114 (−0.599 to 0.371)	0.645	−0.170 (−0.678 to 0.338)	0.510
Cheese	BMI (kg/m^2^)	+0.218 (−0.564 to 0.999)	0.586	+0.613 (−0.278 to 1.505)	0.179
	BAZ (z-score)	−0.011 (−0.377 to 0.355)	0.952	+0.143 (−0.248 to 0.534)	0.475
	HAZ (z-score)	+0.004 (−0.405 to 0.414)	0.983	+0.267 (−0.142 to 0.677)	0.202
Yogurt	BMI (kg/m^2^)	+0.565 (−0.206 to 1.337)	0.152	+0.589 (−0.265 to 1.443)	0.178
	BAZ (z-score)	+0.212 (−0.131 to 0.555)	0.227	+0.154 (−0.211 to 0.520)	0.409
	HAZ (z-score)	−0.100 (−0.454 to 0.253)	0.579	−0.266 (−0.638 to 0.106)	0.163
Butter	BMI (kg/m^2^)	−0.435 (−1.126 to 0.255)	0.218	+0.143 (−0.642 to 0.928)	0.722
	BAZ (z-score)	−0.025 (−0.357 to 0.307)	0.883	+0.075 (−0.250 to 0.401)	0.650
	HAZ (z-score)	−0.111 (−0.465 to 0.243)	0.539	−0.024 (−0.351 to 0.302)	0.884
Lben	BMI (kg/m^2^)	−0.332 (−1.424 to 0.760)	0.552	−0.187 (−0.943 to 0.569)	0.628
	BAZ (z-score)	−0.013 (−0.597 to 0.571)	0.966	−0.082 (−0.405 to 0.242)	0.622
	HAZ (z-score)	+0.155 (−0.332 to 0.643)	0.533	+0.050 (−0.258 to 0.357)	0.751
Jben	BMI (kg/m^2^)	−0.030 (−0.792 to 0.731)	0.938	+0.387 (−0.548 to 1.322)	0.418
	BAZ (z-score)	+0.072 (−0.242 to 0.387)	0.653	+0.171 (−0.202 to 0.544)	0.369
	HAZ (z-score)	−0.237 (−0.606 to 0.132)	0.210	−0.119 (−0.535 to 0.297)	0.575
Raib	BMI (kg/m^2^)	+0.176 (−0.595 to 0.947)	0.655	+0.134 (−0.830 to 1.099)	0.785
	BAZ (z-score)	+0.083 (−0.236 to 0.401)	0.611	+0.163 (−0.217 to 0.543)	0.402
	HAZ (z-score)	+0.006 (−0.334 to 0.347)	0.971	+0.127 (−0.203 to 0.457)	0.451

All models adjusted for: age group, sex, residential area, household size, household income, mother’s education, and father’s education. *β* = unstandardised regression coefficient; 95% CI = 95% confidence interval. Reference = Low consumption (≤1 time/week). Moderate = 2–4 times/week; High = ≥5 times/week. All *p*-values > 0.05. Milk results from the original analysis (Panel A of Tables 3, 4); results for other products from extended analysis.

**Table 4:** Associations between dairy product consumption frequency and binary nutritional outcomes, adjusted logistic regression models (*n* = 248).

Dairy product	Outcome	*n*	Moderate vs. low (ref.)		High vs. low (ref.)		Model
			aOR (95% CI)	*p*	aOR (95% CI)	*p*	
Milk	Overweight/obesity	52	2.705 (0.561 to 13.037)	0.215	3.001 (0.598 to 15.055)	0.182	Standard
	Stunting	12	0.483 (0.074 to 3.142)	0.446	0.315 (0.035 to 2.821)	0.302	Firth^†^
	Abdominal obesity	24	5.088 (0.554 to 683.536)	0.181	4.827 (0.471 to 667.121)	0.219	*Firth†*
Cheese	Overweight/obesity	52	0.764 (0.302 to 1.933)	0.570	1.020 (0.394 to 2.641)	0.967	Standard
	Stunting	12	1.003 (0.365 to 2.754)	0.996	0.741 (0.246 to 2.236)	0.595	Firth^†^
	Abdominal obesity	23	1.850 (0.664 to 5.150)	0.239	2.623 (0.936 to 7.353)	0.067	Firth^†^
Yogurt	Overweight/obesity	52	1.000 (0.433 to 2.306)	1.000	1.339 (0.560 to 3.200)	0.512	Standard
	Stunting	12	1.249 (0.459 to 3.402)	0.663	1.146 (0.383 to 3.427)	0.807	Firth^†^
	Abdominal obesity	23	0.818 (0.261 to 2.559)	0.729	0.503 (0.138 to 1.832)	0.297	Standard
Butter	Overweight/obesity	52	0.544 (0.189 to 1.567)	0.259	1.093 (0.537 to 2.223)	0.806	Standard
	Stunting	12	0.763 (0.227 to 2.566)	0.662	0.870 (0.329 to 2.301)	0.779	Firth^†^
	Abdominal obesity	23	0.719 (0.243 to 2.133)	0.553	0.861 (0.373 to 1.987)	0.726	Firth^†^
Lben	Overweight/obesity	52	0.775 (0.208 to 2.884)	0.703	0.533 (0.241 to 1.180)	0.121	Firth^†^
	Stunting	12	0.969 (0.195 to 4.819)	0.970	0.832 (0.272 to 2.548)	0.748	Firth^†^
	Abdominal obesity	23	1.477 (0.336 to 6.488)	0.605	0.865 (0.309 to 2.422)	0.783	Firth^†^
Jben	Overweight/obesity	52	0.744 (0.254 to 2.176)	0.589	1.601 (0.693 to 3.698)	0.270	Standard
	Stunting	12	1.187 (0.368 to 3.823)	0.774	1.053 (0.341 to 3.246)	0.929	Firth^†^
	Abdominal obesity	23	1.256 (0.431 to 3.661)	0.676	1.142 (0.430 to 3.034)	0.790	Firth^†^
Raib	Overweight/obesity	52	1.137 (0.545 to 2.371)	0.732	1.099 (0.451 to 2.674)	0.836	Standard
	Stunting	12	1.772 (0.671 to 4.682)	0.248	1.040 (0.325 to 3.325)	0.948	Firth^†^
	Abdominal obesity	23	0.937 (0.403 to 2.178)	0.880	1.121 (0.409 to 3.071)	0.824	Firth^†^

All models adjusted for the same covariates as Tables 3, 4. aOR = adjusted odds ratio; *n* = number of children with the outcome. Standard = standard binary logistic regression; Firth^†^ = Firth’s penalised logistic regression applied when total cases < 15 or any cell count < 5. All Hosmer–Lemeshow goodness-of-fit tests: *p* > 0.05. All *p*-values > 0.05. Milk results from the original analysis; results for other products from the extended analysis.

In linear regression models (Panel A), no dairy product showed a statistically significant association with BMI, BAZ, or HAZ after full covariate adjustment (all *p* > 0.05; Table 3). Beta coefficients were consistently small and confidence intervals wide, crossing zero in all cases, suggesting that the absence of significant associations likely reflects limited statistical power rather than a true absence of effect. This pattern was observed for both industrial products (milk, cheese, yogurt) and traditional fermented products (butter, Lben, Jben, Raib).

In logistic regression models (Panel B), no dairy product was significantly associated with overweight/obesity, stunting, or abdominal obesity after adjustment (all *p* > 0.05; Table 4). For stunting (*n* = 9) and abdominal obesity (*n* = 24), the low number of outcome cases required the use of Firth’s penalized logistic regression to ensure stable estimates. Despite this, confidence intervals remained extremely wide for these outcomes across all products, reflecting the very low prevalence of these conditions in the sample and precluding any definitive conclusion.

Overall, no significant independent association was detected between the consumption frequency of any dairy product and any nutritional outcome after full sociodemographic adjustment. These null results are likely attributable to: (1) the use of consumption frequency categories without portion-size data, which prevents dose–response quantification; (2) limited statistical power for low-prevalence binary outcomes (stunting 3.6%, abdominal obesity 9.7%); (3) a ceiling effect for liquid milk, whose very high participation rate (90.3%) reduces the effective contrast between consumption groups; and (4) the highly skewed distribution of traditional product consumption (Lben: 69.4% low; Jben: 72.6% low), which further limits statistical power for these products. Future studies with larger samples and quantitative dietary assessment methods are needed to clarify the specific contribution of individual dairy products to children’s nutritional status in this context.

## 4 Discussion

The present study examined dairy consumption patterns, motivations, and nutritional status in 248 schoolchildren from urban and peri-urban areas of Kenitra Province, northwestern Morocco. Three main findings emerge: (1) modern industrial dairy products, particularly liquid milk, dominate consumption, while traditional fermented products are progressively marginalized; (2) residential area and parental education are the key determinants of traditional dairy consumption; and (3) the urban residential context is associated with better linear growth but markedly higher abdominal obesity prevalence, consistent with a double burden of malnutrition driven by the ongoing nutritional transition; and (4) after full sociodemographic adjustment, no independent association was detected between the consumption frequency of any of the seven dairy products assessed and any nutritional outcome (all *p* > 0.05).

Liquid milk was the most widely consumed product, with 90.3% of children reporting at least moderate participation, a rate consistent with the successful upscaling of industrial dairy processing in Morocco and the progressive displacement of traditional fermented products in urban and peri-urban food systems (16). The high participation rates for cheese (83.1%) and yogurt (76.2%) further reflect the penetration of industrially processed dairy into children’s daily diets, a pattern documented in other North African populations undergoing nutritional transition (9, 10).

Among traditional dairy products, butter presents a distinctive consumption profile. The relatively high proportion of high-level butter consumers (37.9%), coexisting with a sizeable low-consumption group (45.6%), reflects its dual functional role: a culturally embedded ingredient used in Moroccan cooking across all socioeconomic groups, and a traditional food item with strong identity in peri-urban communities. Butter consumption was significantly higher among peri-urban children than among urban children (48.8% vs. 27.2% high consumption; *p* < 0.001; Supplementary Table S1), confirming its characterization as a traditional dietary element that persists in peri-urban environments. This pattern differs from that of Lben and Jben, which are consumed for direct ingestion rather than as a culinary ingredient, likely explaining the divergent frequency profiles between these products despite their shared cultural status as part of Morocco’s artisanal dairy heritage.

In contrast, traditional fermented products, Lben (30.6% participation) and Jben (27.4%), were the least frequently consumed items, with over 69% and 72% of children reporting low or no consumption, respectively. These findings reflect an ongoing dietary transition in Morocco toward industrially processed dairy products, often at the expense of traditional artisanal products (16). However, notable spatial differences were observed in the types of dairy products consumed. Children living in peri-urban areas reported significantly higher consumption of traditional dairy products such as butter, Lben, and Raib compared with those living in urban areas (*p* < 0.001). For instance, 46.3% of peri-urban children reported frequent consumption of Lben, compared with only 7.2% of urban children. This persistence of traditional dairy products in peri-urban environments may be explained by greater proximity to dairy production areas and the continued influence of rural dietary practices (15, 17).

An important observation of this study concerns the motivations underlying dairy consumption. Taste preference emerged as the dominant factor influencing children’s consumption choices, far exceeding perceived health benefits. This suggests that food choices among Moroccan schoolchildren are largely driven by sensory preferences rather than nutritional awareness. Similar patterns have been reported among adolescents in Turkey, where taste preferences and family environment were identified as key determinants of dietary behavior (29). These findings highlight the importance of developing nutrition education strategies that consider behavioral and sensory drivers of food choice when promoting healthy diets among children.

Notably, health-related motivation was reported by 12.8% of urban children versus only 0.8% of peri-urban children. This difference suggests that urban food environments and greater exposure to nutritional education foster greater awareness of the health value of dairy products. This differential has direct implications for intervention design: peri-urban strategies should anchor dairy promotion in sensory appeal and taste enhancement, while urban strategies may leverage health-based messaging more effectively.

The findings also reveal a clear socioeconomic gradient in children’s linear growth. Household income and parental education, particularly that of the father, were significantly associated with height-for-age z-scores (HAZ). Children from households with higher income levels or more educated parents exhibited more favorable growth outcomes. This relationship has been widely documented in low- and middle-income countries (LMICs), where socioeconomic status strongly influences access to high-quality animal protein and essential micronutrients (37, 38). While the “milk hypothesis” posits that dairy consumption supports linear growth through IGF-1-mediated mechanisms (21, 22), and large-scale studies such as the SEANUTS surveys have confirmed a positive association between regular dairy intake and HAZ in Southeast Asian children (24), the regression analyses of the present study did not confirm an independent association between dairy consumption frequency and HAZ after full sociodemographic adjustment (Table 3). These findings suggest that the observed linear growth disparities between urban and peri-urban children are primarily driven by socioeconomic factors, particularly household income and parental education, rather than by dairy intake per se. Conversely, as illustrated in Figure 4, parental education showed consistent inverse gradients for traditional dairy products: higher maternal and paternal education were both significantly associated with lower consumption of Lben (mother: *p* = 0.040; father: *p* = 0.031), Raib (mother: *p* < 0.001; father: *p* < 0.001), and butter (father: *p* = 0.004). These patterns suggest that increasing educational attainment in peri-urban households is associated with a progressive shift away from traditional fermented products, potentially mediating a dietary transition that may reduce exposure to certain traditionally available nutrients. This education-mediated shift, clearly visible in Figure 4, warrants attention in future longitudinal studies. The analysis of residential disparities revealed a striking contrast between urban and peri-urban environments. Children living in urban areas exhibited significantly higher HAZ and BAZ scores than those living in peri-urban areas (*p* < 0.001). These findings suggest that urban environments may offer better access to food resources, healthcare, and diverse diets, thereby reducing the risk of stunted growth. However, this apparent advantage is accompanied by an increased metabolic risk. The prevalence of abdominal obesity was markedly higher among urban children (16.8%) than among peri-urban children (2.4%). This pattern reflects an accelerated nutritional transition in Moroccan cities, where increased availability of ultra-processed foods and more sedentary lifestyles contribute to the rising burden of non-communicable diseases (39, 40). While studies conducted in Bolivia have reported multiple risk factors concentrated in peri-urban areas (15), the present findings suggest that urban environments in Morocco represent a major front line in the emerging childhood obesity epidemic. This finding reinforces the need for targeted prevention strategies in urban Moroccan schools.

Despite the biological plausibility of associations between dairy consumption and nutritional outcomes, through IGF-1-mediated mechanisms for linear growth (HAZ, stunting) and through energy balance for adiposity (BAZ, overweight/obesity, abdominal obesity), the regression analyses conducted in this study did not reveal any statistically significant independent association between the consumption frequency of any of the seven dairy products and any of these outcomes after full covariate adjustment (Tables 3, 4). These null findings are consistent with several recent systematic reviews and meta-analyses indicating that dairy consumption frequency alone, without accounting for portion sizes or overall dietary quality, is insufficient to detect dose–response relationships with nutritional outcomes in children (41–43). Several limitations of the present study require consideration. The cross-sectional design is the most fundamental: it allows us to describe associations but not to establish their direction or rule out reverse causality. Regarding dietary assessment, the FFQ may introduce recall bias, a concern particularly relevant for the younger children in our sample, and, more importantly, captures only the frequency of consumption without any information on portion sizes. The FFQ used in this study was adapted from validated tools used in Moroccan populations (44). This matters more than it might initially appear: classifying a child as a “high” butter consumer and a “high” milk consumer as equivalent exposures ignores the substantially different nutritional implications of these two patterns, especially in terms of saturated fat intake. As a result, a true dose–response analysis between dairy intake and nutritional status was not feasible with the data available, which likely explains in part the null findings reported in Tables 3, 4. Future studies should complement FFQ data with at least two non-consecutive 24-h dietary recalls or weighed food records to allow more precise quantification of nutrient intakes. Finally, the moderate sample size (*n* = 248) limited statistical power for detecting associations with low-prevalence outcomes, a limitation most apparent for abdominal obesity, where the sparsity of events necessitated the use of Firth’s penalized regression and yielded confidence intervals too wide to draw firm conclusions.

Despite these limitations, the study provides valuable and contextually relevant insights into the interplay between socioeconomic factors, residential environment, and dairy dietary habits among Moroccan schoolchildren. Overall, these findings highlight the need for targeted public health interventions adapted to local contexts. In urban areas, strategies should focus on preventing childhood obesity and promoting balanced dietary patterns, whereas in peri-urban areas, efforts should aim to improve linear growth and micronutrient intake. In this context, milk fortification programs, which have already demonstrated effectiveness in reducing anemia in Morocco (34), represent a promising strategy for addressing persistent micronutrient deficiencies among school-aged children (28).

## 5 Conclusion

This study highlights important contrasts in nutritional status and dietary patterns among schoolchildren in northwestern Morocco, emphasizing the influence of residential environment and socioeconomic context on children’s growth and eating behaviors. The findings suggest that the nutritional transition is well underway, with industrial dairy products becoming increasingly predominant, while peri-urban areas continue to maintain elements of traditional dietary practices through the consumption of products such as Lben and Raib. The residential environment appears to be a key determinant of children’s health profiles. Urban environments are associated with better linear growth but also with a higher prevalence of abdominal obesity, reflecting the complex effects of rapid nutritional transition. In contrast, peri-urban areas show lower levels of overnutrition but continue to face challenges related to optimal physical growth.

In addition, the predominance of taste preference as the main motivation for dairy consumption highlights the importance of considering sensory factors when designing nutrition education strategies. Effective public health interventions should therefore combine nutritional awareness with approaches that promote healthy foods while maintaining sensory appeal. Overall, these findings highlight the need for differentiated and context-specific public health strategies aimed at simultaneously addressing persistent undernutrition in peri-urban areas and the increasing burden of childhood obesity in urban environments. Strengthening nutrition education and expanding fortification programs, including within traditional food distribution systems, may represent promising approaches to improving child nutrition and supporting healthy growth in Morocco.

## Data Availability

The raw data supporting the conclusions of this article will be made available by the authors, without undue reservation.
