# Supplementary material for: Dairy consumption, motivations, and nutritional status among schoolchildren in northwestern Morocco: socioeconomic factors and place of residence
Source: Front Nutr. 2026 Jun 17;13:1831350. doi: 10.3389/fnut.2026.1831350 (PMC13322073; doi:10.3389/fnut.2026.1831350)
Supplement: Supplementary file 1 [file Table_1.DOCX]

**Supplementary Table 1.** Frequency of dairy product consumption according to residential area (n = 248).

| **Product** | **Category** | **Total n (%)** | **Urban n (%)** | **Peri-urban n (%)** | **p-value** | **Sig.** |
| --- | --- | --- | --- | --- | --- | --- |
| **Milk** | Low | 24 (9.7) | 9 (7.2) | 15 (12.2) | 0.409 | ns |
|  | Moderate | 140 (56.5) | 73 (58.4) | 67 (54.5) |  |  |
|  | High | 84 (33.9) | 43 (34.4) | 41 (33.3) |  |  |
| **Yogurt** | Low | 59 (23.8) | 30 (24.0) | 29 (23.6) | 0.172 | ns |
|  | Moderate | 114 (46.0) | 51 (40.8) | 63 (51.2) |  |  |
|  | High | 75 (30.2) | 44 (35.2) | 31 (25.2) |  |  |
| **Cheese** | Low | 42 (16.9) | 23 (18.4) | 19 (15.4) | 0.517 | ns |
|  | Moderate | 114 (46.0) | 53 (42.4) | 61 (49.6) |  |  |
|  | High | 92 (37.1) | 49 (39.2) | 43 (35.0) |  |  |
| **Butter** | Low | 113 (45.6) | 74 (59.2) | 39 (31.7) | **<0.001** | ******* |
|  | Moderate | 41 (16.5) | 17 (13.6) | 24 (19.5) |  |  |
|  | High | 94 (37.9) | 34 (27.2) | 60 (48.8) |  |  |
| **Lben** | Low | 172 (69.4) | 114 (91.2) | 58 (47.2) | **<0.001** | ******* |
|  | Moderate | 10 (4.0) | 2 (1.6) | 8 (6.5) |  |  |
|  | High | 66 (26.6) | 9 (7.2) | 57 (46.3) |  |  |
| **Jben** | Low | 180 (72.6) | 88 (70.4) | 92 (74.8) | 0.279 | ns |
|  | Moderate | 31 (12.5) | 14 (11.2) | 17 (13.8) |  |  |
|  | High | 37 (14.9) | 23 (18.4) | 14 (11.4) |  |  |
| **Raib** | Low | 109 (44.0) | 61 (48.8) | 48 (39.0) | **<0.001** | ******* |
|  | Moderate | 73 (29.4) | 54 (43.2) | 19 (15.4) |  |  |
|  | High | 66 (26.6) | 10 (8.0) | 56 (45.5) |  |  |

*Values are n (%) within each residential area category.*

*p-values from Chi-square test. Significance: *** p < 0.001; ns = not significant.*

*Bold p-values and yellow cells: statistically significant (p < 0.05).*

*Shaded rows (grey): first row for each product group.*

*‡ "Low consumption" includes both infrequent consumers and non-consumers (i.e., children who never consume the product).*

**Supplementary Table 2a.** Influence of sociodemographic factors on dairy consumption, Milk, Cheese, Yogurt.

| **Factor** | **Category** | **Milk** | | | | **Cheese** | | | | **Yogurt** | | | |
| --- | --- | --- | --- | --- | --- | --- | --- | --- | --- | --- | --- | --- | --- |
|  |  | **Low ‡** | **Mod** | **High** | **p** | **Low ‡** | **Mod** | **High** | **p** | **Low ‡** | **Mod** | **High** | **p** |
| Total | — | 24 (9.7) | 140 (56.5) | 84 (33.9) |  | 42 (16.9) | 114 (46.0) | 92 (37.1) |  | 59 (23.8) | 114 (46.0) | 75 (30.2) |  |
| Sex | Boys | 14 (10.6) | 74 (56.1) | 44 (33.3) | 0.868 | 17 (12.9) | 66 (50.0) | 49 (37.1) | 0.154 | 36 (27.3) | 61 (46.2) | 35 (26.5) | 0.254 |
|  | Girls | 10 (8.6) | 66 (56.9) | 40 (34.5) |  | 25 (21.6) | 48 (41.4) | 43 (37.1) |  | 23 (19.8) | 53 (45.7) | 40 (34.5) |  |
| Age group | 7–10 yrs | 12 (6.7) | 108 (60.3) | 59 (33.0) | **0.021 *** | 23 (12.8) | 83 (46.4) | 73 (40.8) | **0.013 *** | 37 (20.7) | 85 (47.5) | 57 (31.8) | 0.174 |
|  | 11–14 yrs | 12 (17.4) | 32 (46.4) | 25 (36.2) |  | 19 (27.5) | 31 (44.9) | 19 (27.5) |  | 22 (31.9) | 29 (42.0) | 18 (26.1) |  |
| Res. area | Urban | 9 (7.2) | 73 (58.4) | 43 (34.4) | 0.409 | 23 (18.4) | 53 (42.4) | 49 (39.2) | 0.517 | 30 (24.0) | 51 (40.8) | 44 (35.2) | 0.172 |
|  | Peri-urban | 15 (12.2) | 67 (54.5) | 41 (33.3) |  | 19 (15.4) | 61 (49.6) | 43 (35.0) |  | 29 (23.6) | 63 (51.2) | 31 (25.2) |  |
| HH size | ≤5 | 18 (12.0) | 87 (58.0) | 45 (30.0) | 0.138 | 32 (21.3) | 66 (44.0) | 52 (34.7) | 0.072 | 33 (22.0) | 70 (46.7) | 47 (31.3) | 0.704 |
|  | >5 | 6 (6.1) | 53 (54.1) | 39 (39.8) |  | 10 (10.2) | 48 (49.0) | 40 (40.8) |  | 26 (26.5) | 44 (44.9) | 28 (28.6) |  |
| HH income | Low | 20 (9.9) | 115 (56.9) | 67 (33.2) | 0.878 | 31 (15.3) | 92 (45.5) | 79 (39.1) | 0.240 | 48 (23.8) | 93 (46.0) | 61 (30.2) | 0.999 |
|  | Mid/High | 4 (8.7) | 25 (54.3) | 17 (37.0) |  | 11 (23.9) | 22 (47.8) | 13 (28.3) |  | 11 (23.9) | 21 (45.7) | 14 (30.4) |  |
| Mother edu | Illiterate | 1 (4.3) | 14 (60.9) | 8 (34.8) | 0.926 | 6 (26.1) | 12 (52.2) | 5 (21.7) | 0.727 | 2 (8.7) | 16 (69.6) | 5 (21.7) | 0.104 |
|  | Primary | 14 (10.7) | 72 (55.0) | 45 (34.4) |  | 21 (16.0) | 61 (46.6) | 49 (37.4) |  | 35 (26.7) | 62 (47.3) | 34 (26.0) |  |
|  | Secondary | 8 (9.1) | 50 (56.8) | 30 (34.1) |  | 14 (15.9) | 39 (44.3) | 35 (39.8) |  | 21 (23.9) | 34 (38.6) | 33 (37.5) |  |
|  | University | 1 (16.7) | 4 (66.7) | 1 (16.7) |  | 1 (16.7) | 2 (33.3) | 3 (50.0) |  | 1 (16.7) | 2 (33.3) | 3 (50.0) |  |
| Father edu | Illiterate | 0 (0.0) | 7 (70.0) | 3 (30.0) | 0.205 | 2 (20.0) | 4 (40.0) | 4 (40.0) | 0.186 | 2 (20.0) | 7 (70.0) | 1 (10.0) | 0.456 |
|  | Primary | 18 (12.2) | 85 (57.4) | 45 (30.4) |  | 27 (18.2) | 69 (46.6) | 52 (35.1) |  | 39 (26.4) | 68 (45.9) | 41 (27.7) |  |
|  | Secondary | 1 (2.9) | 16 (45.7) | 18 (51.4) |  | 1 (2.9) | 15 (42.9) | 19 (54.3) |  | 6 (17.1) | 15 (42.9) | 14 (40.0) |  |
|  | University | 5 (9.1) | 32 (58.2) | 18 (32.7) |  | 12 (21.8) | 26 (47.3) | 17 (30.9) |  | 12 (21.8) | 24 (43.6) | 19 (34.5) |  |

**Values:** n (%) within each subgroup category. p-values from Chi-square tests.

*** p < 0.05; ** p < 0.01; *** p < 0.001.** Bold p-values: statistically significant (p < 0.05).

**‡** Low consumption includes both infrequent consumers and non-consumers (children who never consume the product).

HH = household; Res. area = residential area; Mod = Moderate.

Household income recategorized as low vs. middle/high due to very small high-income group.

**Supplementary Table 2b.** Influence of sociodemographic factors on dairy consumption, Butter, Lben, Jben, Raib.

| **Factor** | **Category** | **Butter** | | | | **Lben** | | | | **Jben** | | | | **Raib** | | | |
| --- | --- | --- | --- | --- | --- | --- | --- | --- | --- | --- | --- | --- | --- | --- | --- | --- | --- |
|  |  | **Low ‡** | **Mod** | **High** | **p** | **Low ‡** | **Mod** | **High** | **p** | **Low ‡** | **Mod** | **High** | **p** | **Low ‡** | **Mod** | **High** | **p** |
| Total | — | 113 (45.6) | 41 (16.5) | 94 (37.9) |  | 172 (69.4) | 10 (4.0) | 66 (26.6) |  | 180 (72.6) | 31 (12.5) | 37 (14.9) |  | 109 (44.0) | 73 (29.4) | 66 (26.6) |  |
| Sex | Boys | 61 (46.2) | 23 (17.4) | 48 (36.4) | 0.844 | 91 (68.9) | 3 (2.3) | 38 (28.8) | 0.262 | 103 (78.0) | 16 (12.1) | 13 (9.8) | **0.049 *** | 54 (40.9) | 38 (28.8) | 40 (30.3) | 0.354 |
|  | Girls | 52 (44.8) | 18 (15.5) | 46 (39.7) |  | 81 (69.8) | 7 (6.0) | 28 (24.1) |  | 77 (66.4) | 15 (12.9) | 24 (20.7) |  | 55 (47.4) | 35 (30.2) | 26 (22.4) |  |
| Age group | 7–10 yrs | 82 (45.8) | 29 (16.2) | 68 (38.0) | 0.974 | 124 (69.3) | 9 (5.0) | 46 (25.7) | 0.410 | 131 (73.2) | 20 (11.2) | 28 (15.6) | 0.559 | 77 (43.0) | 55 (30.7) | 47 (26.3) | 0.770 |
|  | 11–14 yrs | 31 (44.9) | 12 (17.4) | 26 (37.7) |  | 48 (69.6) | 1 (1.4) | 20 (29.0) |  | 49 (71.0) | 11 (15.9) | 9 (13.0) |  | 32 (46.4) | 18 (26.1) | 19 (27.5) |  |
| Res. area | Urban | 74 (59.2) | 17 (13.6) | 34 (27.2) | **<0.001 ***** | 114 (91.2) | 2 (1.6) | 9 (7.2) | **<0.001 ***** | 88 (70.4) | 14 (11.2) | 23 (18.4) | 0.279 | 61 (48.8) | 54 (43.2) | 10 (8.0) | **<0.001 ***** |
|  | Peri-urban | 39 (31.7) | 24 (19.5) | 60 (48.8) |  | 58 (47.2) | 8 (6.5) | 57 (46.3) |  | 92 (74.8) | 17 (13.8) | 14 (11.4) |  | 48 (39.0) | 19 (15.4) | 56 (45.5) |  |
| HH size | ≤5 | 74 (49.3) | 21 (14.0) | 55 (36.7) | 0.246 | 116 (77.3) | 3 (2.0) | 31 (20.7) | **0.002 **** | 107 (71.3) | 20 (13.3) | 23 (15.3) | 0.845 | 69 (46.0) | 49 (32.7) | 32 (21.3) | 0.058 |
|  | >5 | 39 (39.8) | 20 (20.4) | 39 (39.8) |  | 56 (57.1) | 7 (7.1) | 35 (35.7) |  | 73 (74.5) | 11 (11.2) | 14 (14.3) |  | 40 (40.8) | 24 (24.5) | 34 (34.7) |  |
| HH income | Low | 85 (42.1) | 35 (17.3) | 82 (40.6) | 0.067 | 134 (66.3) | 9 (4.5) | 59 (29.2) | 0.097 | 153 (75.7) | 22 (10.9) | 27 (13.4) | 0.063 | 86 (42.6) | 53 (26.2) | 63 (31.2) | **0.002 **** |
|  | Mid/High | 28 (60.9) | 6 (13.0) | 12 (26.1) |  | 38 (82.6) | 1 (2.2) | 7 (15.2) |  | 27 (58.7) | 9 (19.6) | 10 (21.7) |  | 23 (50.0) | 20 (43.5) | 3 (6.5) |  |
| Mother edu | Illiterate | 11 (47.8) | 4 (17.4) | 8 (34.8) | 0.161 | 14 (60.9) | 0 (0.0) | 9 (39.1) | **0.040 *** | 15 (65.2) | 5 (21.7) | 3 (13.0) | 0.203 | 8 (34.8) | 4 (17.4) | 11 (47.8) | **<0.001 ***** |
|  | Primary | 51 (38.9) | 27 (20.6) | 53 (40.5) |  | 83 (63.4) | 5 (3.8) | 43 (32.8) |  | 91 (69.5) | 21 (16.0) | 19 (14.5) |  | 63 (48.1) | 27 (20.6) | 41 (31.3) |  |
|  | Secondary | 48 (54.5) | 8 (9.1) | 32 (36.4) |  | 69 (78.4) | 5 (5.7) | 14 (15.9) |  | 70 (79.5) | 4 (4.5) | 14 (15.9) |  | 37 (42.0) | 38 (43.2) | 13 (14.8) |  |
|  | University | 3 (50.0) | 2 (33.3) | 1 (16.7) |  | 6 (100.0) | 0 (0.0) | 0 (0.0) |  | 4 (66.7) | 1 (16.7) | 1 (16.7) |  | 1 (16.7) | 4 (66.7) | 1 (16.7) |  |
| Father edu | Illiterate | 5 (50.0) | 4 (40.0) | 1 (10.0) | **0.004 **** | 3 (30.0) | 1 (10.0) | 6 (60.0) | **0.031 *** | 6 (60.0) | 2 (20.0) | 2 (20.0) | 0.605 | 1 (10.0) | 0 (0.0) | 9 (90.0) | **<0.001 ***** |
|  | Primary | 63 (42.6) | 27 (18.2) | 58 (39.2) |  | 104 (70.3) | 6 (4.1) | 38 (25.7) |  | 112 (75.7) | 16 (10.8) | 20 (13.5) |  | 66 (44.6) | 42 (28.4) | 40 (27.0) |  |
|  | Secondary | 12 (34.3) | 2 (5.7) | 21 (60.0) |  | 20 (57.1) | 2 (5.7) | 13 (37.1) |  | 27 (77.1) | 3 (8.6) | 5 (14.3) |  | 13 (37.1) | 9 (25.7) | 13 (37.1) |  |
|  | University | 33 (60.0) | 8 (14.5) | 14 (25.5) |  | 45 (81.8) | 1 (1.8) | 9 (16.4) |  | 35 (63.6) | 10 (18.2) | 10 (18.2) |  | 29 (52.7) | 22 (40.0) | 4 (7.3) |  |

**Values:** n (%) within each subgroup category. p-values from Chi-square tests.

*** p < 0.05; ** p < 0.01; *** p < 0.001.** Bold p-values: statistically significant (p < 0.05).

**‡** Low consumption includes both infrequent consumers and non-consumers.

HH = household; Res. area = residential area; Mod = Moderate.

Household income recategorized as low vs. middle/high due to very small high-income group.
